# Novel Insights into the Development and Function of Cilia Using the Advantages of the *Paramecium* Cell and Its Many Cilia

**DOI:** 10.3390/cells4030297

**Published:** 2015-07-29

**Authors:** Junji Yano, Megan S. Valentine, Judith L. Van Houten

**Affiliations:** Department of Biology, University of Vermont, Burlington, VT 05405, USA

**Keywords:** *Paramecium*, cilia, calcium, signal transduction, swimming behavior, electrophysiology, proteomics

## Abstract

*Paramecium* species, especially *P. tetraurelia* and *caudatum*, are model organisms for modern research into the form and function of cilia. In this review, we focus on the ciliary ion channels and other transmembrane proteins that control the beat frequency and wave form of the cilium by controlling the signaling within the cilium. We put these discussions in the context of the advantages that *Paramecium* brings to the understanding of ciliary motility: mutants for genetic dissections of swimming behavior, electrophysiology, structural analysis, abundant cilia for biochemistry and modern proteomics, genomics and molecular biology. We review the connection between behavior and physiology, which allows the cells to broadcast the function of their ciliary channels in real time. We build a case for the important insights and advantages that this model organism continues to bring to the study of cilia.

## 1. Introduction

**Figure 1:**
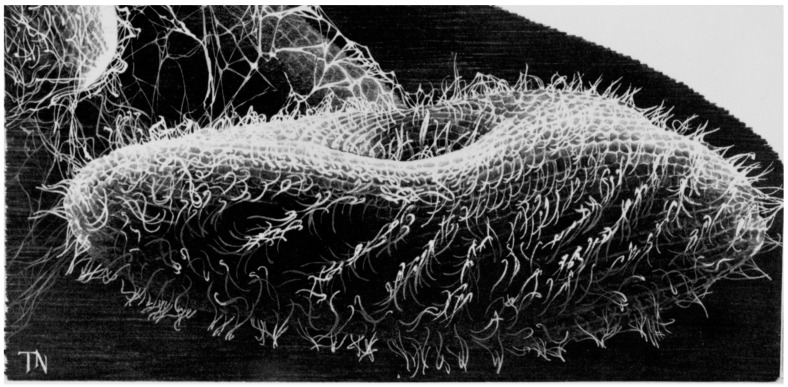
*Paramecium tetraurelia* showing the waves of cilia beating toward the posterior of the cell. From Grass Calendar, 1985.

Ciliates have been studied for their ciliary motility and sensory functions for over 100 years [1]. Among ciliates, *Paramecium sp.* have served as an excellent model system for the development and function of cilia, in part because of their large numbers of cilia [2]. *P. tetraurelia* and *P. caudatum* in particular stand out for their contributions to cilia biology through electrophysiological studies that can be combined with behavior, biochemistry, proteomic and genomic studies of wild type and mutant cells. Small changes in ciliary beating, which is determined by the membrane electrical properties, are amplified as easily observable changes in swimming behavior. Therefore, one can monitor the active physiology of the cell and ciliary channels by watching the cells swim, which is easily done with a dissecting scope because of the cells’ large size (>100 μm × >50 μm). A depiction of this beautiful cell and its cilia is shown in Figure 1. Kung capitalized upon this connection of physiology and behavior to generate and select mutants that allowed for a genetic dissection of *Paramecium* behavior [3]. The mutants have led to a more complete understanding of the channels of the ciliary membrane and some surprises about the nature of their control. 

In addition to these advantages, the repeating pattern of basal bodies and cilia on the cell surface has made it possible to identify ciliary defects and misalignments that would be very difficult to find in primary ciliary systems, which typically have one cilium per cell. The thousands of cilia present on each *Paramecium* cell allow for the isolation and collection of these organelles for biochemical studies [4,5]. Such approaches are not as simple using cells containing a single cilium. The cilia of *Paramecium* are both motile and sensory, containing a “9+2” microtubule organization, with nine outer doublets and a central pair. Most primary non-motile cilia are sensory in function, lacking the central pair of microtubule doublets (reviewed in [6]). Both types of cilia are important for sensory functions, and many of the receptors and channels necessary for chemoresponse are located in the ciliary membrane of *Paramecium* [7,8,9,10]. These attributes of *Paramecium* have laid the ground work for current molecular and proteomic analyses of its cilia (see [11,12] for new perspective on *Paramecium*). 

## 2. The Advantages of having an Excitable Membrane: *Paramecium* Broadcasts the Activity of Its Ciliary Ion Channels through Its Behavior

**Figure 2:**
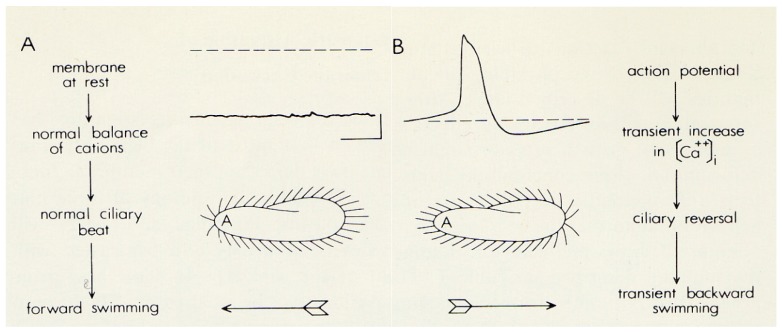
A. These images are to demonstrate that the intracellular membrane potential of *Paramecium* is negative (about −25 to −40 mV); the corresponding ciliary beat is toward the posterior of the cell and the cell swims forward. B. In depolarizing solutions, like high K^+^ or Ba^2+^, the cell’s membrane potential depolarizes and reaches threshold for the action potential. During the action potential calcium enters the cilia through voltage gated channels, the high levels of Ca^2+^ change the power stroke of the cilia, which now beat most strongly toward the anterior and move the cell backward. The action potential is quickly over and the calcium is removed from or sequestered in the cilia, allowing the ciliary beat and swimming to return to normal. With permission from Science [3].

*Paramecium* cells beat their thousands of cilia toward the posterior of the cell in waves that propel the cell forward along a helical path. This path changes with the cell’s membrane potential. The helix tightens and the cell swims faster with hyperpolarization; with depolarization, the cells slow down and the helix widens. These changes are brought about by changes in the ciliary power stroke and the degree of alignment of the power stroke with the cell’s posterior (elegant work of Naitoh, Kaneko, Eckert, and Machemer reviewed in [13,14,15,16]). If the depolarization is sufficient, this little swimming neuron [17,18] will activate the voltage gated calcium channels (Ca_(v)_), which are exclusively in the cilia [10,19], to create a regenerative depolarization (action potential). The elegant work of Naitoh, Kaneko, Eckert, and Machemer reviewed in [13,14,15,16] determined that the Ca^2+^ entering through Ca_(v)_s during the action potential interacts with the axoneme to reverse the power stroke of the cilia, sending the cellbackward (Figure 2).

Because the reversal is temporary, the cell soon swims forward in a new, almost randomly determined direction [20]. The backward swimming ends with a reduction in calcium at the axoneme from µM back to resting nM concentrations [21]. The Ca^2+^ from the Ca_(v)_ conductance is sequestered by proteins like calmodulin and removed from the cilia by calcium pumps [22]. Calcium also feeds back on the channels to inactivate the Ca_(v)_s. 

Ca^2+^ is necessary and sufficient to reverse the power stroke of the cilia, as shown by classic experiments in which cells were made permeable with detergent [16,23,24]. These permeabilized cells swim forward if provided with Mg^2+^-ATP, and reverse their swimming if Ca^2+^ is also provided at or above µM levels, suggesting that the Ca^2+^ interacts directly with the axoneme to change the beat. Lieberman and others also demonstrated that these permeabilized cells even retain the metachronal wave as in Figure 1 when induced to swim backward [24].

There are two kinds of K channels of the cilia that repolarize the cell [15] to bring the membrane potential back to rest after the action potential is initiated. The fast activating K_(v)_ channel is opened with depolarization, while the slower activating K_(Ca)_ channel is calcium dependent. 

The ability to generate these action potentials sets *Paramecium* apart from other cells that are not “excitable.” The action potential allows the cell to react immediately to situations like bumping into an object or swimming into an area of high salt or repellents [25]. What links these stimuli together is that they all cause depolarizations sufficient to elicit action potentials. 

## 3. Cilia are Signal Transduction Compartments 

**Figure 3:**
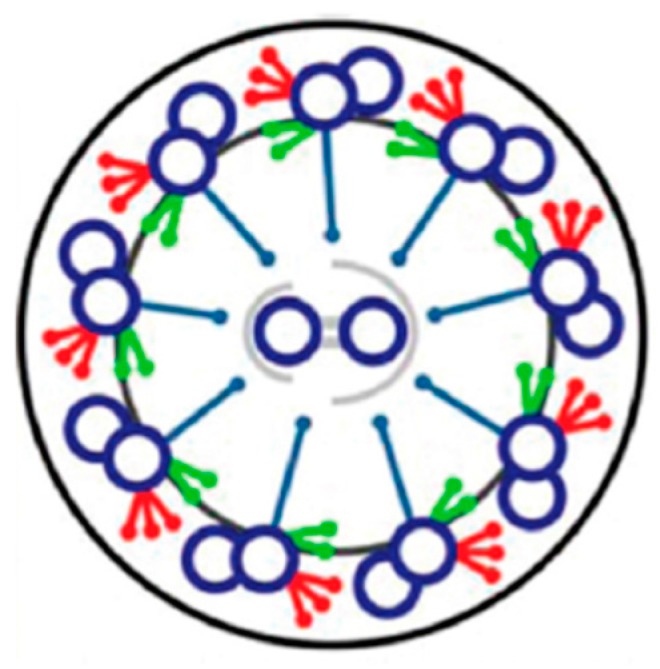
Diagram of a cross section of a cilium. Outer arm dyneins in red; inner arm dyneins in green; doublets of microtubules in blue; radial spokes connecting central pair to outer doublets. Adapted from [30], with permission.

Within the ciliary membrane is the elegant axoneme that is composed of nine outer doublets of microtubules that extend the length of the cilium with two central asymmetrical doublets that are connected to the outer ones by radial spokes (Figure 3). Dynein ATPase arms, which are attached along the length of the outer microtubules, move the B tubules of the doublets on one side of the cilium toward the minus end, forcing the doublets to slide relative to each other and forcing the cilium to bend [26]. When dyneins are active on the opposite side of the cilium, the bend direction changes. The dyneins are classified as Outer and Inner Dynein Arms, for their relative proximity to the cilium surface or interior. The Outer Dynein Arms (ODA) affect power and beat frequency; the Inner Dynein Arms (IDA) generate the ciliary bends and the shape of the ciliary waveform (reviewed in [27]).

**Figure 4:**
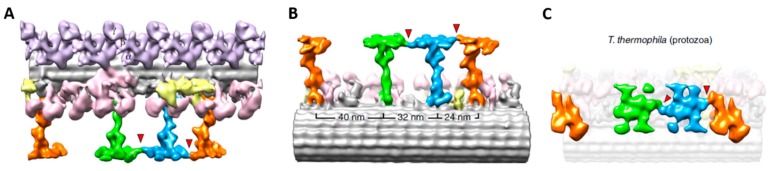
Tomogram from a *Tetrahymena* cilium. Isosurface renderings show the averaged 96-nm axonemal repeats of the Radial Spoke proteins. Radial spoke proteins 1, 2, and 3 are shown in green, blue, and orange, respectively. The ODA are shaded in purple, IDA are shaded in light pink, and yellow shading indicates the Nexin-Dynein Regulatory Complex. The red arrowheads indicate the connections between the radial spokes. (**A**) is a longitudinal-front view, (**B**) longitudinal back view, and (**C**) a bottom view. Adapted from [28], with permission.

The advent of tomography has made it possible to visualize the elegant details of the ciliary structure, specifically the radial spokes in relation to their repeated positions along the outer double microtubules (Figure 4) [28]. The reversal of the power stroke of the cilia causes the reversal of swimming direction; this maneuver requires changes in the interactions among the central pair, the radial spokes and the inner dynein arms. There are signals (Ca^2+^ and cyclic nucleotides) generated in the cilia upon changes in membrane potential that orchestrate the changes in dynein function and ciliary beat frequency and form. The kinases and phosphatases that communicate the signals to the dyneins are part of the axonemal structure [27,29]. The *Paramecium* model has been useful in sorting out the signaling steps. 

## 4. Depolarization and Signal Transduction in the Ciliary Compartment

Both depolarization and hyperpolarization of *Paramecium* initiate second messengers and signal transduction pathways that affect the function of axonemal proteins. The initiation of second messengers results in altered ciliary beat form. To effectively and efficiently control the axonemal proteins and ciliary beat, the protein content of the ciliary membrane is different than that of the cell body. A perfect example of this is in the case of *Paramecium*. In depolarization, the second messenger is intraciliary Ca^2+^ from the Ca_(v)_ channels that are located exclusively in the ciliary membrane [10,19]. The ciliary Ca_(V)_ channels are responsible for *Paramecium*’s action potential. Thus, the cilium of *Paramecium* acts as a Ca^2+^ signaling compartment with a protein complement that is separate from the cell body membrane. 

In neuronal cells, surface membrane protein composition must differ between presynaptic and postsynaptic membranes for neurotransmission. Similarly with cilia, the membrane proteins that determine its sensory and motility functions are mostly distinct from the cell body membrane proteins. Studies utilizing direct recording from mammalian primary cilia [31,32] show that the mammalian primary cilium, as in the case of *Paramecium* cilia, is a special Ca^2+^ signaling compartment isolated from the rest of the cell. Thus, the Ca^2+^-permeable channels, such as the PKD2-dependent channels in mammalian primary cilia [33] and the voltage-gated Ca^2+^ channels in *Paramecium,* function within a special Ca^2+^-signaling organelle [34]. 

Also within this special Ca^2+^ compartment are the K^+^ channels that repolarize the membrane potential to resting level and appear to be mostly, if not exclusively, in the cilia [35]. The Ca^2+^ that activates the K_(Ca)_ channel has been shown to come from the Ca_(v)_ channels of the cilia [36]. Husser and workers [37] demonstrated that there is no spillover of Ca^2+^ from the ciliary action potentials into the cell body, further demonstrating that the Ca^2+^ that activates K_(Ca)_ comes from the ciliary Ca_(v)_.

While other depolarization- or Ca^2+^-activated channels are not exclusive to the cilia, the proteins present in the ciliary Ca^2+^ compartment are the ones that participate in controlling ciliary motion following the initiation of action potentials. For example, duration of backward swimming is a function of the activity of cilia-limited Ca_(v)_ channels, the repolarizing K^+^ conductances, and other Ca^2+^-dependent channels that conduct sodium ions (Na^+^) or magnesium ions (Mg^2+^). These channels can prolong the plateau of the action potential and increase the duration of backward swimming. Because there is no spillover of Ca^2+^ into the cell body to activate plasma membrane channels, the Ca^2+^ that activates these Na_(Ca)_ and Mg_(Ca)_ conductances must derive from the Ca_(v)_ channels of the cilia. Although these Na_(Ca)_ and Mg_(Ca)_ channels might not be exclusively in the cilia, it appears that only those in the cilia affect the duration of backward swimming initiated by the action potential. 

The protein that is the likely channel for the Mg_(Ca)_ in *Paramecium* is Polycystic Kidney Disease protein 2 (PKD2) which is located in both the cilia and cell membrane [38]. In humans, PKD2 is a critical Transient Receptor Potential Polycystin family (TRPP) channel that, if defective, will lead to autosomal dominant polycystic kidney disease (ADPKD). PKD2 associates in kidney tubule cells with polycystin-1 (PKD1) that is similar to a TRP protein, but does not function as a channel. Flow through the kidney tubules activates the mechanosensory PKD1 and PKD2 complex, allowing Ca^2+^ to flow into the kidney cells, keeping the cells from proliferating. ADPKD can be traced back to the failure of kidney primary cilia to sense the mechanical stimulus from flow through tubules because the conductance of Ca^2+^ through ciliary PKD2 protein fails [39,40]. 

As mentioned, the *P. tetraurelia* ortholog of PKD2 resides in the cilia and plasma membrane, and, like the mammalian counterpart, requires a very specific protein trafficking route to reach the cilium and a second route to reach the plasma membrane [9]. However, in contrast to mammalian PKD2 that conducts Ca^2+^, PKD2 in *P. tetraurelia* appears to conduct Mg^2+^ because when PKD2 is reduced in *P. tetraurelia*, the Mg^2+^-induced prolonged backward swimming behavior and depolarization are concomitantly reduced [38].

A reduction in *P. tetraurelia* PKD2 produces a phenocopy of the XntA mutant [38], which has lost the Mg_(Ca)_ conductance that prolongs the action potential in Mg^2+^ solutions and lost the ability to swim backward in Mg^2+^ [41,42]. XntA has some structural similarities to PKD1 but does not appear to be the Mg^2+^ channel [41]. The XntA mutant phenotype can be mostly reversed to wild type with excess PKD2. Immunoprecipitations suggest that PKD2 and XntA interact with each other (directly or indirectly) in both the ciliary and cell membranes. However, the effects of this association are site-specific. When XntA and PKD2 are together in cilia, XntA appears to temper Mg^2+^-induced behavior and depolarization. Removal of cilia removes this inhibitory effect which can be measured as Mg^2+^-induced depolarization probably due to the PKD2 and XntA proteins that still interact in the cell membrane. Even deciliated XntA mutant cells show robust depolarizations in Mg^2+^ as long as PKD2 is available [38]. 

An additional intraciliary second messenger pathway dependent upon depolarization by the voltage gated Ca^2+^ channels, described by Schultz and workers, is an increase in intraciliary cyclic GMP [43]. Others established that cyclic GMP (and cyclic AMP, see below) affected the ciliary beat of detergent-treated cells and sheets of cilia that are both accessible to bath solutions [44,45]. Cyclic GMP did not reverse the beat in these preparations, but, depending on the preparation, increased or decreased beat frequency. It is clear, however, that cyclic GMP antagonized the effects of Ca^2+^ to reverse ciliary beat, leading some to speculate that cyclic GMP helps the recovery of the forward beat [23]. The signal pathway components including guanylyl cyclase, Protein Kinase G (PKG), protein phosphatases and phosphodiesterases are all integral to the cilia, hinting at roles in modifying radial spoke, central pair and dynein functions by post-translational modifications. However, the substrates of the kinase and phosphatases remain undefined.

As discussed above, the *Paramecium* made permeable to their extracellular milieu suggest that Ca^2+^ and not another second messenger acts directly upon the axonemes to reverse the ciliary beat during depolarization. Preston and Saimi [23] argue from analysis of the timing of the reversal compared to signaling pathways, that Ca^2+^ acts directly upon an axonemal component to reverse the power stroke. The protein interactions that mediate the Ca^2+^ dependent reversal are not known, but there are candidates among the calmodulin binding proteins of the axoneme that are discussed below. 

## 5. Hyperpolarization and Signal Transduction in the Ciliary Compartment

Hyperpolarization in *Paramecium* is clearly linked to fast forward swimming due to cilia beating more frequently and more toward the posterior [46,47]. As with a neuron, a reduction in extracellular K^+^ in the *Paramecium* cell’s bath solution will hyperpolarize the cell. Likewise, some attractant chemical stimuli cause cells to hyperpolarize [48,49]. Both of these methods of hyperpolarizing cells are coupled with fast swimming and a very rapid increase in cyclic AMP [50,51,52,53]. Cyclic AMP applied to the ciliated sheets of *Paramecium* surface modifies the ciliary beat direction and frequency providing a demonstration of the fast swimming upon hyperpolarization [44,54,55]. Similarly, Bonini and Nelson showed increases in beat frequency in permeabilized cells with cyclic AMP [56]. 

The connection between hyperpolarization and adenylyl cyclase activation to account for the increased cyclic AMP became more clear when Schultz and workers purified an adenylyl cyclase that was activated by a K^+^ conductance [51,52]. The subsequent cloning of the genes for adenylyl cyclase helped to explain the regulation of the cyclase by a K^+^ conductance. All sixteen adenylyl cyclase genes in *P. tetraurelia* appear to code for proteins of this same structure of a cyclase domain linked to a K^+^ channel domain [52]. This physical linkage between these two functional domains could explain the rapid K^+^ conductance upon stimulation of the cyclase and the association of increases in intracellular cyclic AMP with stimuli that hyperpolarize the cell [51]. It is not clear how the domains of the enzyme communicate, but the association of cyclic AMP and hyperpolarization is clear [56]. The adenylyl cyclase may also be responsible for the coupling of the hyperpolarization with rapid cyclic AMP production when paramecia are stimulated with the attractant glutamate [50], whose receptors are limited to the cilia [38,57]. 

Adenylyl cyclases are found in both the *Paramecium* cell body and ciliary membrane. A proteomics analysis enabled determining which ones were specific to the cilia (see below) [58]. Other proteins of the cyclic AMP signaling pathway (such as PKA, phosphatases PP1 and PP2A, A kinase anchor protein (AKAP)) are associated with key proteins of the *Chlamydomonas* axoneme that affect the wave form and beat of the cilia (reviewed in [27,29]). The signal pathway components are positioned within the axoneme to affect the interactions of the central pair apparatus and radial spokes with the inner dyneins to control ciliary waveform and with the outer dyneins to affect velocity of sliding [27,54]. In *Chlamydomonas*, a radial spoke functions as an AKAP that positions PKA near the inner dynein arms [27]. Some of the puzzle pieces of the substrates of the signaling kinases are falling into place, such as the 29 kD axonemal protein of the 22S dynein outer arm that co-purifies with an inner arm dynein and a substrate of cyclic AMP-dependent phosphorylation in *Paramecium* [44,55,56]. This 29 kD protein regulates swimming speed and therefore accounts for at least part of the mechanism by which hyperpolarization and increased ciliary cyclic AMP accelerate ciliary beating through increased microtubule translocation. While *Paramecium* axonemal proteins that are substrates of PKA or PKG have been identified [44,55,59,60], their roles in ciliary regulation are not entirely clear. The Ca^2+^ sensor has yet to be identified in *Paramecium*. 

## 6. The Advantage of Mutants

Preston observed that *Paramecium* broadcasts the activity of its ion channels through its swimming behavior [61]. This broadcast makes it easy for the observer to intuit the ion channel activity from the cells’ swimming speed, turning frequency, and duration of backward swimming during the turn. This connection between behavior and channel activity inspired Kung to carry out a genetic dissection of swimming behavior that led to many useful mutants. The best known of these mutants are the Pawns, named for the chess piece because they cannot move backward for lack of a functional ciliary Ca^2+^ channel [3,61,62]. These mutants, called Pawn in *P. tetraurelia* and Caudatum Non-Reversal (CNR) in *P. caudatum* [63] (reviewed in [23]), have been valuable in studies of ion conductances that can be observed in the absence of the ciliary Ca_(v)_ conductance in these cells [64]. Only Ca_(v)_ conductance is affected in Pawn; the K^+^ channels of these mutants are normal [65]. Analysis of the *P. caudatum* CNR mutants made it possible to identify centrin as a player in the regulation of the Ca_(v)_ channels [66]. 

Successful function of the Ca_(v)_ channels in cilia of *P. tetraurelia* is dependent upon the small Pawn proteins. Without these proteins, the cells cannot swim backward or generate an action potential because they lack the Ca_(v)_ conductance [3], as is the case with deciliated cells [19]. Lodh [67] showed that Pawn mutants have no Ca_(v)_ channels in their ciliary membrane unless the missing wild type gene was introduced into the cells to rescue the wild type phenotype. 

Other mutants shed light on the control of the K_(Ca)_ channel that is critical for repolarizing the membrane and the Ca^2+^-activated Na^+^ (Na_(Ca)_) channel that, when open, prolongs the action potential and causes backward swimming [68]. Mutations in two distinct regions of the same gene (for calmodulin) are responsible for drastically different behavioral phenotypes [69]. Without the normal sequence of the C terminal lobe of calmodulin, the K_(Ca)_ channel fails to repolarize the cell leading to long backward swimming following an action potential in mutants called Paranoiacs. Without the normal sequence of the N terminal lobe of calmodulin the Na_(Ca)_ channel is not activated, causing other mutants called Fast-2 to fail to sustain the long backward swimming in Na solutions [69]. 

Many other behavioral mutants were isolated and characterized, making it possible to analyze every conductance known in *Paramecium* with the help of mutants with fascinating names like Paranoiac, Chameleon, Dancer, Restless, and Eccentric [42,61,62,70]. 

## 7. Depletion of Ciliary Proteins 

The sorting of proteins into and out of the cilia is determined by the transition zone between the basal body and cilium [71]. Also critical to developing and maintaining a functional cilium are the processes of trafficking proteins to the surface for incorporation into the axoneme and membrane of the cilium. *Paramecium* shares the critical proteins for this filtering and trafficking with other systems, but *Paramecium* stands out as a model to study ciliary channel and signaling proteins. For example, PKD2 depends upon the coat complex of Bardet-Biedl Syndrome (BBS) proteins for proper location in the ciliary membrane [72]. Seven BBS proteins along with BBIP10 combine to form a coat complex, important for the trafficking of proteins to the ciliary membrane [73]. Together with Rabin8 and the GTPase Rab8, certain ciliary proteins such as the somatostatin receptor protein 3 (SSTR3) [74] and PKD2 [9,72] require this coat complex to reach the cilia. In *Paramecium*, both PKD2 and a calcium-activated K^+^ channel (SK1a) are lost from the cilia of cells when the BBS complex proteins are depleted [9]. In contrast, the *P. tetraurelia* ciliary Ca_(v)_ channels reach the ciliary membrane without these BBS proteins, or upon the accessory subunits such as those that guide mammalian Ca_(v)_ channels to their membrane locations [75]. 

A distinct advantage of *Paramecium* is its repeating pattern of units on its surface from which one or two cilia emanate. This means that basal bodies and their cilia are arranged in neat rows from posterior to anterior. The subsurface rootlets that anchor cilia likewise run in stereotypical rows. When there is a defect in the organization of cilia on the surface, the deviation from the normal pattern broadcasts the problem, much as behavior broadcasts abnormal ciliary physiology. This structural broadcast is not possible in cells with one primary cilium per cell. 

**Figure 5:**
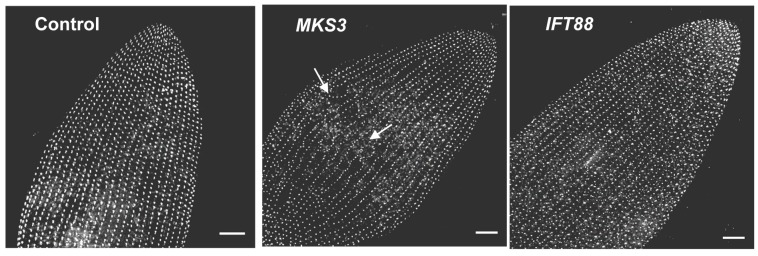
Cells were stained using anti-centrin to visualize the basal bodies. Images represent stacks of Z sections, approximately 10 µm thick. The images shown are the dorsal surface of the anterior end of the cell. Basal bodies should be arranged in organized rows, as seen in the control and *IFT88* depleted cells. These latter cells are controls for the effects of shortened and lost cilia because they are depleted in a protein that is important for the import of proteins into the cilia. The *MKS3* depleted cell (center panel) shows the basal bodies not aligned and no longer in straight rows (white arrows) at the midline of the cell. Scale bars: 10 µm. With permission from *Cilia* [9].

The advantage of this highly organized and repetitive surface is highlighted in our study of the conserved transition zone protein meckelin (MKS3). Reduction of MKS3 by RNA interference (RNAi) results in the global shortening and loss of cilia in *P. tetraurelia*, probably due to problems with the transition zone to pass proteins into the cilia for maintenance [76]. However, there are additional phenotypes of this MKS3 reduction: lines of basal bodies are out of alignment (Figure 5). It appears that the basal bodies and their associated rootlets have wandered off course [76]. This phenotype is very striking, but would be difficult to identify with one cilium per cell.

## 8. Ciliary Membrane Proteomics

We have attempted to establish that a *Paramecium* cilium is a special compartment for the membrane and signal transduction proteins that control ciliary beat frequency and wave form. It is estimated from ciliary proteomics and gene expression during cilia regeneration that the *Paramecium* cilia comprises around 1100 proteins. Not all of these ciliary proteins can be sorted out using mutations or depletion techniques such as RNAi [77]. To make things more complex, the *Paramecium* genome has undergone three duplications [78], meaning that there are many pairs and triplets of genes. Some of these genes have retained their original function while others have lost function or taken up new functions. Therefore, it is not sufficient to infer the proteome of the *Paramecium* cilium from the genomics of the cilium even though some genomics tools are quite good [79,80]. Proteomic analysis using high accuracy mass spectrometry is essential to identify proteins that reside in the cilia without the bias of Western blotting techniques. 

Two advantages of ciliates like *Paramecium* for examining the proteins of cilia are that there are thousands of cilia covering each cell and these cilia can be cleanly removed for use in biochemical analysis [81]. (The cell body membranes can also be prepared for biochemistry or for electrical recording [10,19].) The large number of cilia on *Paramecium,* compared to generally one primary cilium per cell*,* is advantageous for proteomic analysis of ciliary proteins. It is especially important to have sufficient starting materials to identify channels and other membrane proteins that we have found to be of low-abundance [58].

The *Paramecium* ciliary proteome is estimated from the genomic analysis ParameciumDB and gene expression during cilia regeneration is estimated to comprise around 1100 proteins [82,83]. Our interest is in the membrane proteins that we know from others’ analyses of cilia will differ from the membrane proteome of other (somatic) plasma membrane domains [84]. The transmembrane proteins should include receptors and ion channel involved in receiving chemical and mechanical stimuli from the environment and in modifying the signal within the cilium to change swimming behavior in response to stimuli. Also important to identify are the signal transduction components (kinases, phosphatases, phosphodiesterases, cyclases) that allow for depolarization and hyperpolarization to modify axoneme function and ciliary beat. 

In order to concentrate the membrane proteins of the cilia, we isolated cilia and further separated them into ciliary membrane and axonemes [58] as previously described [5]. We further treated the ciliary membrane with Triton-X114 for phase separation in order to concentrate transmembrane and lipidated proteins into the detergent phase [58,85]. This detergent treatment also had the beneficial effect of cleaving the Glycosylphosphatidyl inositol (GPI)-anchor of the peripheral proteins that are inserted into the outer leaflet of the membrane by this anchor [86,87]. The cleaved GPI-anchored proteins partitioned into the aqueous phase, allowing us to analyze the detergent soluble proteins in their absence. Since two thirds by mass of the surface proteins are GPI-anchored proteins [88], separating them into the aqueous phase allowed us to concentrate the low abundance trans-membrane proteins in the detergent phase for mass spectrometry. 

Our mass spectrometric analyses of cilia, ciliary membrane and ciliary membrane detergent phase show that membrane proteins makeup about 10% of the total proteins. Of proteins identified from the detergent phase of Triton X-114 phase separation, 55% are the membrane proteins, which contain proteins with predicted transmembrane domains or proteins associating with the membrane through lipid moiety (ion channels, ion pumps, adenylyl cyclase, Ca^2+^ dependent protein kinases and phosphatase, and Rab GTPases). 

**Figure 6:**
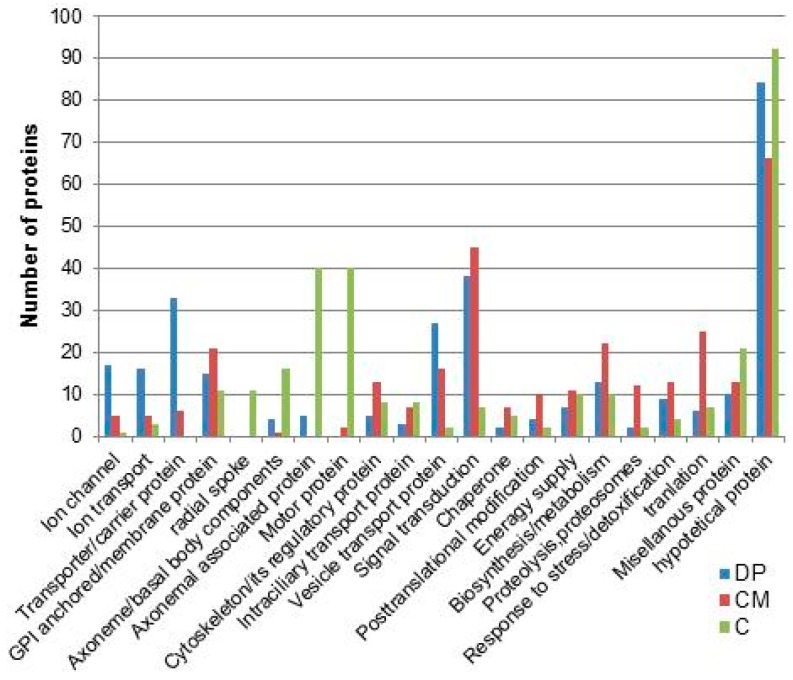
Frequency of proteins organized by function from the 300 most abundant proteins from each preparation: non-fractionated cilia (C), ciliary membrane (CM), and the detergent phase of Triton X-114 phase separation (DP). With permission from Elsevier [58].

Figure 6 demonstrates that the trans-membrane proteins of interest to us for their function in ciliary motility and sensory response are enriched in the detergent phase of Triton X-114 phase separation. Note that the signal components that we have discussed in this review are prevalent among the proteins in the detergent phase (blue in Figure 6) and that structural proteins are prevalent in the whole cilia (green Figure 6). Mass spectrometry allowed us to identify the three voltage gated calcium channels from among at least 40 known in the genome that are expressed in the cilia. Our study allowed us to determine that a particular small conductance Ca^2+^-activated K^+^ channel (SK1a) and a Mg^2+^-channel like exchanger (XntA) really are in the cilia. Likewise, we were able to determine which adenylyl cyclase is localized in the cilia for control by hyperpolarization. The presence of these proteins in cilia has been validated using RNAi, tagged protein expression, and immunoprecipitation [9,52,58]. 

One of our interests is in the mechanism of removal of Ca^2+^ that enters through the Ca_(v)_ channels. It is thought to bind to calmodulin and be removed by plasma membrane calcium ATPase pumps (PMCA) [22]. Our proteomic analysis of the transmembrane proteins shows that several PMCAs are in the ciliary membrane, but, judging from peptide numbers, PMCA18 and 19 are the most enriched [58]. (Indeed, our undergraduate course entitled “Biology 205: Advanced Genetics” that analyzes *Paramecium* ciliary membrane by mass spectrometry consistently finds these PMCAs, although the Ca_(v)_ is extremely difficult to find.) To validate this finding, we performed RNA interference (RNAi) for PMCA18 and 19 and found prolonged backward swimming, which indicates that ciliary Ca^2+^ remains high without the activity of these pumps [89]. We also used epitope tagged VGCC1c in ciliary membrane to show that this Ca_(v)_ channel immunoprecipitates with PMCA18 and 19 (confirmed by mass spectrometry [89]). 

The co-localization of the Ca_(v)_ and PMCA in the cilium is intriguing to us. The ciliary channel conducts very few Ca^2+^ molecules in order to achieve a µM concentration and reverse ciliary beat [21]. Close proximity of the Ca_(v)_ and PMCA in cilia could provide that tight regulation of the Ca^2+^ entering the cilium. Since *Paramecium* cilia are separate lipid membrane compartments as well as protein and Ca^2+^ compartments [90], it is possible that association of the PMCAs and channels could be facilitated by lipid rafts. Indeed, the phase separation that we use to enrich for membrane proteins also enriches for proteins that reside in lipid rafts and PMCAs in other systems are known to be in lipid rafts [91,92]. The trypanosome flagellum is enriched in lipid rafts, and lipid rafts appear to play a role in localizing proteins, including a calcium sensor, to their flagella [93]. 

The next level of proteomic analysis of *Paramecium* cilia will be analysis of post-translational modifications of the signaling pathways that implement the depolarization and hyperpolarization signaling to change the ciliary beat.

## 9. Summary

*Paramecium* species, especially *P. tetraurelia* and *caudatum*, provide a broad and deep foundation for research. The confluence of genetics, behavior, and electrophysiology brought us a new understanding of ciliary motility. Structural studies, biochemistry, genomics, and molecular biological studies followed with important insights into the key protein and second messenger players in the control of ciliary beating. All of these insights have come together as we use mass spectrometry in an unbiased way to identify and confirm the proteins involved in ciliary beat control.
